# A ribose world: current status and future challenges of plant RNA biology

**DOI:** 10.1093/jxb/erad070

**Published:** 2023-04-09

**Authors:** Sebastian Marquardt, Pablo Andrés Manavella

**Affiliations:** Department of Plant and Environmental Sciences, Copenhagen Plant Science Centre, University of Copenhagen, Bűlowsvej 21A, 1870 Frederiksberg, Denmark; Instituto de Agrobiotecnología del Litoral (CONICET-UNL), Facultad de Bioquímica y Ciencias Biológicas, Universidad Nacional del Litoral, 3000 Santa Fe, Argentina

**Keywords:** Epigenetics, mRNA, RNA stability, small RNAs, splicing, transcription

**Elucidation of the cellular functions of RNAs represents one of the most exciting research areas in modern life sciences. RNAs play crucial roles in plant biology, including developmental regulation and environmental responses. Advances in sequencing technologies have benefitted the genome-wide analysis of RNA modifications, the resolution of RNA secondary structures, the mapping of epigenetic modifications, the identification of RNA-edited sequences, and the discovery of novel RNA classes. These advances have allowed impressive progress in our understanding of cellular roles of RNAs, but have also given rise to new questions (** **Manavella *et al.*, 2023** **). In this special issue, we have collected expert contributions that provide updates on different aspects of plant RNA biology, thereby highlighting the most intriguing open questions and the future direction of this exciting field of research.**

## RNA production

RNA polymerases synthesize multiple distinct RNA species in cells. RNA is synthesized from either DNA templates by DNA-dependent RNA polymerases (RDPs) or RNA templates through RNA-dependent RNA polymerases (RDRs). Five nuclear RDP polymerase (NRP) complexes (NRPA–E) generate different types of RNAs from genomic DNA with functional roles across the cell (Wierzbicki *et al.*, 2021). The genomes of grasses contain a sixth copy of the largest nuclear RDP subunit (Trujillo *et al.*, 2018), suggesting that an additional nuclear RNA polymerase complex (NRPF) awaits biochemical characterization. Plant cells contain two additional genomes in the chloroplasts and mitochondria where separate DNA-dependent RNA polymerases are active. In mitochondria, two phage-like single-peptide polymerases are active (RPOTm and RPOTmp). In plastids, we know of at least three DNA-dependent RNA polymerases, namely plastid encoded polymerase (PEP) that represents a multi-subunit enzyme reminiscent of bacterial polymerases, plus two phage-like single-peptide polymerases (RPOTp and RPOTmp) (Liere *et al.*, 2011).

RDRs generate double-stranded RNAs (dsRNAs) from single-stranded RNA (ssRNA) templates. Cellular infection strategies of plant pathogens may rely on intermediates. dsRNAs are a potent trigger for cellular pathogen defence pathways of plant cells, namely RNA interference. The Arabidopsis genome contains six RDRs linked to the silencing of distinct target RNAs (Willmann *et al.*, 2011), but the copy number varies across plant species (Krishnatreya *et al.*, 2021). RDR activity can also start from ssRNA, where it can be primed by 22-nucleotide small RNAs (sRNAs), resulting in secondary sRNA production from an initial sRNA trigger for the cellular amplification of silencing (Lopez-Gomollon and Baulcombe, 2022). RDR activity primed by sRNAs exemplifies a common theme in RNA biology during gene expression: RNA contributes to cellular RNA production and gene expression.

## Roles of RNA during gene expression

R-loops represent DNA–RNA structures where RNA invades the DNA double-strand (Zheng *et al*., 2023). R-loops associated with RDPs tend to be near transcription start sites (TSSs) and DNA regions where transcription terminates (de Felippes and Waterhouse, 2023). However, trans-acting nuclear non-coding RNAs (ncRNAs) can invade the DNA double-strands to bind target loci, thus creating R-loops with effects on gene expression through epigenomic remodelling. R-loops in plants have been shown to be genome-wide and genetic tools exist to stabilize and resolve them, thus promising rapid progress in elucidating their roles. Intriguingly, R-loops also mark sites of DNA damage and they promote genome stability; however, it remains to be determined whether these roles are linked to NRP activity resulting in ncRNAs, such as is seen in DNA damage in humans.

Splicing offers a key mechanism to shape the expression of distinct messenger RNA (mRNA) isoforms from the DNA sequences of single eukaryotic genes (Tognacca *et al*., 2023). Splicing occurs at nuclear RNA polymerase II (NRPB) transcripts through the activity of the spliceosome complex (Marquardt *et al.*, 2023). The spliceosome contains proteins and small nuclear RNA (snRNA) components that are critical for activity. Splicing thus illustrates how some RNAs play critical roles for the expression of other RNAs (snRNAs and mRNAs in this case). Plants diversify their proteomes through the regulation of splicing (Tognacca *et al*., 2023). Introns removed through splicing promote the expression of transgenes in plants (Rose, 2018), perhaps indicating how splicing could help to distinguish plant RNAs from extracellular RNAs targeted by RDRs.

DNA sequences in plant genomes that elicit transcription termination and 3´-end formation represent ’terminators’ (de Felippes and Waterhouse, 2023). Similar to splicing, alternative termination and alternative poly-adenylation (APA) of mRNA generate different isoforms of mRNAs (Marquardt *et al.*, 2023). Recent data highlighting APA near TSSs resulting in short promoter-proximal RNAs (sppRNAs) illustrate large-scale regulation of mRNA expression through promoter proximal termination (Thomas *et al.*, 2020). From the perspective of plant biotechnology, terminators are a crucial component of successful transgene expression (de Felippes and Waterhouse, 2023). Inefficient termination of RNA polymerase II (Pol II) transcription results in RNAs that serve as substrates for RDRs, resulting in dsRNA formation and transgene silencing. Future research into plant RNA 3´-end formation and termination promises to generate key knowledge for engineering transgene expression vectors with reduced risk of silencing.

The sequences of RNAs in plant cells can differ from their genomic DNA template sequence. A key reason for differences in base-pairs are RNA-modifying enzymes. RNA editing illustrates how the sequences of transcribed RNAs differ from their DNA templates at specific nucleotide positions (Knoop, 2023). RNA editing is common in plant organelles and represents a fascinating example of co-evolution between the RNA maturation processes in organelles and the necessary co-factors encoded by the nuclear genome. RNA editing can occur in coding sequences and can be necessary to obtain a fully functional protein; however, RNA editing sites are also located in non-coding parts of RNAs where the functional roles of the editing events are less clear.

RNA helicases represent an important class of enzymes that facilitate the association and disassociation of RNAs with other cellular molecules (Li *et al*., 2023). They are thus central to many cellular functions of RNAs, including splicing of nuclear and organellar transcripts, R-loop resolution, micro RNA (miRNA) production, transcriptional termination, and targeting RNAs for degradation. Loss-of-function genetics of RNA helicases in Arabidopsis has identified many essential genes, highlighting the key roles of regulating RNA functions through helicases. Mutations in RNA helicases involved in substrate targeting of the nuclear exosome RNA degradation complex (Lange and Gagliardi, 2022) facilitates detection of many nuclear ncRNA species as a starting point for functional dissection (Thomas *et al.*, 2020).

Cellular proteins that bind and act on RNAs intersect with pathogenic effector proteins that suppress cellular defence mechanisms (Lopez-Gomollon and Baulcombe, 2022). The suppression of cellular RNA interference (RNAi) by effector proteins encoded by pathogenic viruses and bacteria highlights the multiple interfaces for effector proteins to reduce the efficiency of cellular RNA-based defense pathways. While it is clearly intuitive to recognize the benefit for RNA viruses, DNA viruses such as geminiviruses rely on the transcription and translation machineries of the infected plant cells (Wang and Lozano-Durán, 2023). Geminivirus effector proteins facilitate the transcription of the nuclear viral genome into mRNAs. Of particular note are viral transcripts that are undergo splicing. Introns are unusual in the small genome of viruses, arguing for the possibility that geminiviral effector proteins might intersect with splicing to amplify the protein-coding mRNA isoforms generated from small viral genomes. In agreement with this idea, the geminiviral coat protein co-localizes with components of the exon junction complex (Wang and Lozano-Durán, 2023).

Examination of the cellular localization and abundance of RNAs can help in determining their functions. Single-molecule RNA fluorescent immune-hybridization (smRNA FISH) uses a fluorescent probe to label single RNA molecules in cells, and the technique thus offers information on the cellular RNA localization and expression levels. In this special issue, Duncan *et al*. (2023) validate the use of four mRNAs as reference controls for smRNA FISH experiments in Arabidopsis roots. Their definition of ‘reference mRNAs’ promises to facilitate the technical implementation of this method in laboratories without prior experience, thereby making the benefits of smRNA FISH in providing the sub-cellular localization and quantification of RNAs much more easily accessible for researchers of RNA biology.

Transcription of genes by Pol II into mRNA provides the substrate for ribosomal translation into proteins. Ribosomes are composed of protein and ncRNA subunits critical for translation. Cellular pathways control the journey of mRNAs from the nucleus to the cytoplasmic ribosomes, and the ribosomal activity. These mechanisms of cellular regulation offer an explanation for the differences that can exist between protein levels in cells estimated based on mRNA expression. Ribosomes engaged in translation are purified from cells in polysome fractions, and the association of mRNAs in polysomes thus offers insights into their translation. Here, Bai *et al*. (2023) highlight the dynamics of mRNA translation into proteins during seed development. They integrate mRNA expression data and polysome occupancy data to obtain information on the interplay between gene expression and translation during this vital stage in the life cycle. A key resource that promises to inform future research in this area is a web-based interface to mine the data in the seed translation network (the ‘SeedTransNet’). In a case study, the authors identify the translational regulator PM19L as key node for the coordination of seed maturation. Advances such as these promise to give insights into RNA biology during seed development, with implications for food production.

The multiple cellular roles of RNAs offer tantalizing avenues for biotechnological exploitation. Included in this special issue is a study that highlights the advantages and opportunities of RNA biotechnology in the context of agriculture (Rodriguez *et al*., 2023). Current solutions for pest control using pesticides and crop engineering to produce genetically modified organisms (GMOs) have met resistance from consumers. Directing cellular RNAi towards pathogens using exogenous application of dsRNAs offers a promising GMO-free approach to increase resistance without pesticides. The efficient, large-scale delivery of dsRNAs currently represents a barrier in plant RNA nanotechnology, but the successful application of exogenous RNA holds the promise of future benefits for agricultural production, including modularity, fast time-scales, and scalability.

## The hidden word of non-coding RNAs

The idea of ‘information flow’, which describes how DNA is decoded into proteins using an intermediary called mRNA, is at the core of molecular biology and a well-known concept throughout education. However, there is a big pool of cellular RNAs that are functional but do not encode proteins. Thanks to the development of methods to sequence RNAs, thousands of theses non-coding RNAs have now been identified, annotated, and studied. Indeed, the study of ncRNAs has become one of the central topics within RNA biology research in most organisms. In plants, studying these molecules has not only pursued an understanding of their fundamental properties and mechanisms of action, but also how we can take advantage of them with biotechnological uses. In this issue, Gullotta *et al*. (2023) discuss the functional effects of ncRNAs on plant biology in relation to crop breeding and agricultural traits. Their review focuses on well-characterized ncRNAs associated with processes of assisted crop improvement. It also provides an overview of the naturally occurring variation in the non-coding genome and the impact such polymorphisms have on the functional effects of ncRNAs.

The most abundant and relevant types of ncRNAs are commonly divided into four categories, transfer RNAs (tRNAs), ribosomal RNAs (rRNAs), long non-coding RNAs (lncRNAs), and small RNAs (sRNAs) (Eddy, 2001). The study of tRNA and rRNA started after they were first identified more than 45 years ago, and new discoveries and unexpected functions are still being found (Schimmel, 2018). tRNAs are well known for their essential role during protein synthesis; However, other functions are now attributed to these macromolecules in all living organisms. In this issue, Chery and Drouard (2023) present an updated overview of these additional functions. These include, but are not limited to, the involvement of tRNAs in tetrapyrrole biosynthesis, mRNA stabilization and transport, priming reverse-transcription of viral RNAs, and regulating gene expression through the production of small non-coding RNAs named tRNA-derived RNAs (tDRs).

lncRNAs are a class of ncRNAs comprising molecules with a length greater than ~200 nt that do not encode for stable proteins (Wierzbicki *et al.*, 2021). The catalog of RNA molecules assigned to this subclass of ncRNAs continues to increase through high-throughput sequencing of RNAs (Ivanov *et al.*, 2021).However, the functions of many of these molecules, if any, is largely unknown. We understand the biological roles of far fewer lncRNAs than mRNAs. The roles of well-characterized lncRNAs in regulating processes such as transcription, chromatin remodeling, splicing, protein localization and translation, and also their potential biotechnological applications are extensively reviewed in this issue by Yang *et al*. (2023).

The category of sRNAs includes several subfamilies including miRNAs, small interfering RNAs (siRNAs), small nucleolar RNA (snoRNAs), small nuclear RNAs (snRNAs), and the recently discovered tiny RNAs (tiRNAs) (Baldrich *et al.*, 2019). Among these, the miRNAs are the most studied class. These molecules are encoded in the plant genome as a lncRNA known as primary miRNA (pri-miRNA). Pri-miRNAs are then processed into mature miRNAs either during their transcription or in the nucleoplasm by the Type III RNase DICER-Like 1 (DCL1) (Gonzalo *et al.*, 2022). In this issue, Mencia *et al*. (2023) discuss the most recent discoveries in the biogenesis of miRNAs in plants and the most compelling open questions on this topic. The precise production of miRNAs is critical to maintaining plant homeostasis, mainly by controlling development and orchestrating responses to environmental stresses. For many years, this has made miRNAs an appealing biotechnological tool to silence genes of interest. However, manipulating or using miRNAs to change given features involves transgenic plants, which in the case of crops involves legislative and public perception barriers. The manipulation of miRNAs for biotechnological purposes has gained new interest with the development of genome editing tools. For example, miRNA-mediated gene-silencing can now be released by removing endogenous miRNAs using CRISPR/Cas9 editing. Here, Ferreira and Reis (2023) provide a viewpoint paper where they argue that gene editing of miRNA target sites is preferable over deletion of the miRNA genes. According to the authors, this approach avoids affecting the expression of secondary miRNA target genes, thereby allowing the specific manipulation of selected genes.

Different from miRNAs, which are always encoded by *MIRNA* genes, many alternative sources of dsRNAs can give rise to siRNAs. The source of the dsRNA, the extension of complementarity between strands, and the subcellular localization dictate which DCL protein process them into siRNA and the characteristics of the mature siRNAs (Borges and Martienssen, 2015). For example, DCL3 processes perfectly complementary nuclear dsRNAs into siRNA of 24 nt in length, while cytoplasmic dsRNA derived from exogenous viruses are commonly processed by DCL2 and DCL4 to produce siRNAs of 22 nt and 21 nt, respectively. Among the many classes of siRNA existing in a cell, the most abundant are those of 24 nt produced by DCL3 from transposon-derived RNA polymerase IV (Pol IV) transcripts. Through a pathway known as RNA-directed DNA methylation (RdDM), these siRNAs act as guardians of the genome integrity, ensuring that transposable elements (TEs) are kept under control (Matzke and Mosher, 2014).

Independently of their nature, both miRNAs and siRNAs are both loaded into argonaute (AGO) proteins to form RNA-induced silencing complexes (RISCs) to fulfill their regulatory actions. How a miRNA or siRNA will act largely depends on which AGO protein loads them. All plants encode multiple copies of AGO proteins with different preferences for the length, the 5’-nucleotide, and the subcellular distribution of the sRNA. Each of these AGOs will later act differently depending on its nature and the loaded sRNA (Ma and Zhang, 2018). For example, while AGO1 prefers to load 21-nt miRNAs to silence genes post-transcriptionally by destabilizing the targeted mRNAs, AGO4 loads 24-nt siRNAs to trigger DNA methylation, mostly at transposons and repetitive elements to silence genes at the transcriptional level. Here, Martin-Merchan *et al*. (2023) provide a comprehensive updated review of our current knowledge regarding AGO proteins, in which they discuss the key features of AGO domain architecture, the evolution of this family of proteins, their expression patterns and subcellular localization, and the biological functions of the 10 AGO proteins in Arabidopsis.

Interestingly, many ncRNAs, especially the sRNAs, can exit one cell and act autonomously in another. This movement and cross-cellular action are particularly relevant during development to create the gene expression gradients required for the proper formation of tissues and organs. Such movement is also crucial for the systemic defense against pathogens, especially viruses. But the most impressive recent discovery has been that of the capacity of certain organisms to interact with each other, both parasitically and symbiotically, using extracellular RNA molecules (exRNAs) (Clarke *et al.*, 2019; Cai *et al.*, 2021). In this issue, Borniego and Innes (2023) review the RNA species that make up the extracellular RNAome and discuss putative mechanisms that could account for the diversity of exRNAs. They also discuss the potential functions of vesicular and extravesicular exRNAs, especially during interactions between plants and pathogens, in intercellular communication, and in other physiological processes.

## Perspectives

This special issue highlights many of the multiple roles RNA species play in plants. The world of RNAs in plant cells is complex. While many are needed for gene expression and protein production, they are also critical for cellular resilience, and yet they also provide a platform for successful infection strategies by pathogens. Strikingly, after decades of RNA research, the purpose of most ncRNAs produced in cells remains to be determined. RNAs are moving into public perception, but they also literally move within cells between and across the whole plant. Entire pathways are dedicated to regulating the cellular activities of RNAs, indicating that there are clearly advantages for cells in relying on RNA-based regulation. Elucidating the molecular principles underlying this RNA-based regulation in plants and the benefits RNA-based solutions offer to plant cells for solving environmental challenges during growth promises to inspire biotechnological solutions for current barriers in agriculture.
